# Predictors and Potential Clinical Implications of Residual Postoperative Pleural Space After Uniportal-Vats Lobectomy

**DOI:** 10.3390/jcm14144988

**Published:** 2025-07-15

**Authors:** Maria Letizia Vita, Antonio Giulio Napolitano, Adriana Nocera, Claudia Leoni, Arianna Gallo, Khrystyna Kuzmych, Leonardo Petracca-Ciavarella, Maria Teresa Congedo, Elisa Meacci, Filippo Lococo, Stefano Margaritora, Dania Nachira

**Affiliations:** Department of Thoracic Surgery, Fondazione Policlinico Universitario “A.Gemelli” IRCCS, Università Cattolica del Sacro Cuore, 00168 Rome, Italy; marialetizia.vita@policlinicogemelli.it (M.L.V.); antoniogiulionapolitano@gmail.com (A.G.N.); leoniclaudia.98@gmail.com (C.L.); ariannagallo96@gmail.com (A.G.); kkristina.kuz@gmail.com (K.K.); leonardo.petraccaciavarella@policlinicogemelli.it (L.P.-C.); mariateresa.congedo@policlinicogemelli.it (M.T.C.); elisa.meacci@policlinicogemelli.it (E.M.); filippo.lococo@policlinicogemelli.it (F.L.); stefano.margaritora@policlinicogemelli.it (S.M.); dania.nachira@policlinicogemelli.it (D.N.)

**Keywords:** pleural space, lobectomy, uniportal vats

## Abstract

**Objectives:** Residual postoperative pleural space (RPPS) is a common event after pulmonary lobectomy. Uniportal video-assisted thoracoscopic surgery (VATS) lobectomy has been associated with a higher incidence of RPPS. This study aims to evaluate the incidence, the predictors, and potential clinical implications of RPPS following Uniportal VATS lobectomy. **Methods:** Patients who underwent Uniportal VATS lobectomy, without any previous neoadjuvant treatment, from June 2016 to March 2020, were retrospectively analyzed. RPPS was assessed using the last chest X-Ray prior to discharge and measured by Collins method (%). **Results:** Among 492 patients who underwent Uniportal VATS lobectomy, 325 (66.1%) developed RPPS. The mean RPPS volume measured by the Collins method was 15.46 ± 8.59% (vs. Collins = 4.2% in no-PRPS). An RPPS > 10.5% of Collins was significantly associated with a higher risk of postoperative air leak (AUC: 0.69, sensitivity: 69%, specificity: 54%, *p* < 0.001). Multivariable analysis identified the following predictors of RPPS > 10.5%: right-sided surgery (*p* < 0.001), upper lobectomy (*p* = 0.01), and prolonged air leak (*p* = 0.003). Patients with RPPS had a higher risk of only radiologically visible postoperative subcutaneous emphysema on the final chest X-ray (*p* = 0.041) and were more frequently discharged with a chest tube connected to a Heimlich valve (*p* < 0.001). Within 90 days post-discharge, 24 (4.9%) patients were readmitted due to increased RPPS (1.4%, requiring drainage in 5 cases [1%]), progression of subcutaneous emphysema (1.6%), and pleural effusion (1.8%, requiring drainage in 6 cases [1.2%]). However, RPPS was not associated with an increased overall risk of postoperative complications (*p* = 0.31) or 90-day readmission (*p* = 0.43). **Conclusions:** RPPS is a common occurrence following Uniportal VATS lobectomy but is not associated with clinically significant complications. The current study findings identified BMI, active smoking, right-sided surgery, and prolonged air leak as significant predictors of RPPS.

## 1. Introduction

Residual postoperative pleural space (RPPS) is defined as an empty space visible on radiological examinations after lung resections. It is a relatively frequent condition following pulmonary lobectomy, with a reported incidence of up to 40% of cases [1,2]. Inefficient pleural drainage, restrictive lung diseases, extensive parenchymal resections, prolonged air leaks, and mediastinal fixation have been identified as risk factors [1,2] for the development of RPPS.

Usually, RPPS is a benign and self-limiting condition whose natural course leads either to complete obliteration of the space or to the persistence of a stable space with no clinical consequences [3]. In most cases, the space is typically filled with sterile fluid; subsequently, thoracic physiological adaptations—such as compensatory lung emphysema of the remaining lung, mediastinal shift, diaphragmatic elevation, and narrowing of intercostal spaces—contribute to the gradual resolution of the RPPS.

Conversely, the simultaneous presence of a persistent air leak and increased pleural thickness may be associated with an unfavorable outcome [3]. Indeed, the lack of apposition between the two pleural surfaces may delay air leak closure, create a non-sterile environment, and increase the risk of intrathoracic infection. The longer this complication persists, the greater the likelihood of significant adverse events, including prolonged hospitalization and a negative impact on both healthcare costs and patient quality of life [4].

Among all minimally invasive approaches, Uniportal VATS appears to be associated with an increased risk of RPPS after lobectomy. However, although several methods have been proposed in the literature [5,6,7] to reduce both the incidence and the extent of RPPS after Uniportal VATS lobectomy, no study has accurately analyzed the phenomenon or measured the actual volume of RPPS. And above all, no data are currently available on the potential clinical implications of RPPS in the postoperative period. Quantifying RPPS with the Collins method allows for an objective stratification of patients based on pleural space volume. This may help in anticipating prolonged air leaks, guiding decisions about chest tube removal, and planning the intensity of post-discharge surveillance.

Therefore, the main aims of this study are to evaluate the incidence, predictive factors, and potential clinical implications of RPPS in patients undergoing Uniportal VATS lobectomy.

## 2. Materials and Methods

### 2.1. Ethical Statement

This is a monocentric, retrospective observational study, approved by our Ethics Committee (Università Cattolica del Sacro Cuore, Rome) in October 2020 (Protocol ID:3553) and therefore performed in accordance with the ethical standards of the Declaration of Helsinki and its later amendments. All patients signed an informed consent for their participation in the study and for anonymous treatment of their clinical data.

The Strengthening the Reporting of Observational Studies (STROBE) checklist was followed for reporting of data and results in this study.

The present study reports all the results of patients enrolled between June 2016 and March 2020.

The inclusion criteria were adult patients who provided signed informed consent and underwent Uniportal VATS lobectomy for early-stage Non-Small Cell Lung Cancer (NSCLC) or large and perihilar resectable metastases from other primary tumors with disease under local control.

The exclusion criteria were open surgery or conversion to thoracotomy during surgery, any previous adjuvant treatment, any other associated lung or chest-wall resections, bilobectomies, and segmentectomies.

### 2.2. Surgery and Post-Operative Management

All pulmonary lobectomies were performed under general anesthesia with single-lung ventilation in Uniportal VATS [8], the standard approach used at our center since 2016. At the end of surgery, all patients received an intercostal nerve block performed by operating surgeons under thoracoscopic guidance [8], according to the standard analgesic protocol at our center.

As usual in this approach, the chest tube was inserted through the same incision in the 4th or 5th intercostal space at the mid-axillary line [5,8]. The drainage was removed on postoperative day 2 or 3, provided there was no evidence of air leak and the amount of pleural effusion was less than 500 mL/day. Otherwise, patients were discharged with the chest tube connected to a Heimlich valve and re-evaluated as outpatients for eventual drainage removal. In general, suction is not routinely used after a lobectomy at our center. It is applied only in cases of important air leakage with clinically significant subcutaneous emphysema or symptomatic increasing postoperative pneumothorax on chest X-ray, in which case a suction of −20 cmH_2_O is applied for about 48 h. In cases of prolonged air leakage (>5 days), if the air leak is not severe enough to require surgical aerostasis revision, the chest tube is connected to a Heimlich valve, allowing for patient discharge if clinically stable and eupnoic in room air, without supplemental oxygen.

#### RPPS Evaluation

The RPPS was evaluated on postoperative chest X-rays performed before chest tube removal (usually on the second or third postoperative day after lobectomy). RPPS was quantified using the Collins method, which is generally applied for quantification of pneumothorax side [9].

**Figure 1:**
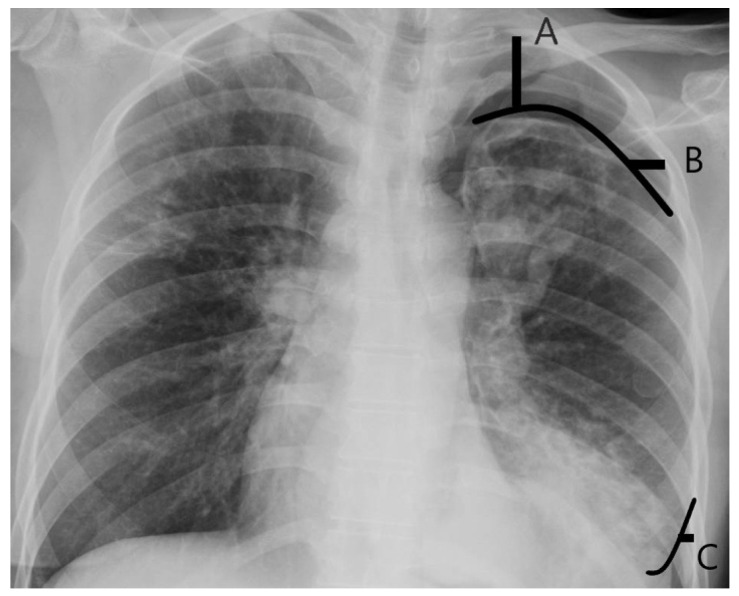
Collins method. A is the maximum apical interpleural distance, B is the interpleural distance at midpoint of upper half of lung, C is the interpleural distance at midpoint of lower half of lung.

The Collins formula used for measuring RPPS (%) was (Figure 1):

RPPS (%) = 4.2 + [4.7 × (A + B + C)],

where:

A is the maximum apical interpleural distance, 

B is the interpleural distance at the midpoint of the upper half of the lung, 

C is the interpleural distance at the midpoint of the lower half of the lung. All distances are measured in centimeters.

Therefore, in case of complete collapse of the residual lung after surgery and negative RPPS, the Collins index is 4.2%.

The main clinical, surgical, and radiological variables analyzed per each patient included gender, age, smoking habits, body mass index (BMI), chronic obstructive pulmonary disease (COPD), any cardiovascular disease, American Society of Anesthesiologists (ASA) score, side of surgery, type of lobectomy, pulmonary function (PaO_2_, FEV1%, FVC%, DLCO), RPPS, chest drainage duration, hospital stay, discharge with Heimlich valve, complications, and 90-day readmission, etc.

### 2.3. Primary and Secondary Outcomes

The primary outcome of the study was to evaluate the incidence of RPPS after Uniportal VATS lobectomy.

The secondary outcomes were to identify the predictors for RPPS and assess its potential clinical implications after Uniportal VATS lobectomy.

### 2.4. Statistical Analysis

Categorical variables were reported as numbers (%) and continuous variables as mean ± standard deviation if normally distributed or median and interquartile range (IQR) if not normally distributed. The Kolmogorov–Smirnov test was used to evaluate the normal distribution of data.

Categorical variables were compared by the Chi-squared test or Fischer’s exact test; continuous variables by the independent-sample Student’s *t*-test or the Mann–Whitney U-test, if normally or non-normally distributed.

Univariable and multivariable regression models were used to assess the main risk factors associated with RPPS.

After assessing the main post-operative complication in our series (prolonged air leakage for more than 5 days), a ROC Curve analysis was performed to assess the cut-off point that provided the best predictor value of RPPS (measured by the Collins index) associated with prolonged post-operative air leakage.

Univariable and multivariable regression analyses were also performed to assess the main risk factors associated with an RPPS > cut-off point associated with prolonged air leakage.

Only variables with a *p* < 0.2 in the univariable analysis were included in the multivariable regression analysis.

A *p* < 0.05 was considered statistically significant.

Statistical analysis was performed using the IBM SPSS Statistics for Macintosh, Version 25.00 (Armonk, NY, USA).

## 3. Results

Among 2440 patients who underwent lung resections at our center between June 2016 and March 2020, only 492 met the inclusion and exclusion criteria.

The main clinical and pathological characteristics of these 492 patients who underwent Uniportal VATS lobectomy are summarized in Table 1.

Two hundred and fifty-five patients were male (51.8%), with the mean age being 68.62 ± 9.05 years. In 301 (61.2%) cases the operation was performed on the right side, and 255 (51.8%) patients underwent upper lobectomy. The mean tumor diameter was 2.46 ± 1.41 cm.

A total of 325 patients (66.1%) developed an RPPS of measurable extent on the two projections of the final chest X-ray obtained before chest tube removal. In no case was the RPPS clinically significant enough to require the insertion of a second chest tube or to delay the patient’s discharge or tube removal (in the absence of air leakage).

The mean entity of the apical RPPS, evaluated in terms of intercostal spaces between the posterior rib arches on the chest X-Ray, was 1.53 ± 1.39 (median: 2). Fifteen patients (4.6%) had a basal RPPS and 29 (8.9%) a lateral one. Overall, the mean RPPS measured by Collins method was 15.46 ± 8.59%. In patients without RPPS at chest X-Ray, the Collins index was 4.2%, as expected according to the formula cited above [10].

The main types of postoperative complications included prolonged air leak (more than 5 days) in 48 cases (9.8%), atelectasis in 17 (3.5%), atrial fibrillation in 20 (4.1%), pneumonia in 6 (1.2%), and subcutaneous emphysema in 25 (5.1%). Some patients experienced more than one complication.

Among the patients with prolonged postoperative air leak (>5 days), 6 (1.2%) required reoperation for surgical revision of aerostasis, while 8 (1.6%) underwent a bedside talc-slurry pleurodesis.

In the absence of an air leak, the chest tube was removed after 4.66 ± 2.82 days, with a mean hospital stay of 4.81 ± 2.33 days.

In 78 cases (24.0%), the patients were discharged with a chest tube connected to a Heimlich valve due to persistent air leak or high fluid output (>500 mL/24 h) and were monitored and re-evaluated weekly as outpatients for chest tube removal. The chest tube was removed after a median of 7 days post-discharge, once air leak cessation was confirmed for at least 24 h and fluid amount was less than 500 mL/day. The presence of a stable RPPS on chest X-Ray did not prevent chest tube removal.

At univariable analysis, the main predictors of RPPS were weight (*p* = 0.018), BMI (*p* = 0.002), active smoking (*p* = 0.048), right-sided surgery (*p* = 0.003), and prolonged postoperative air leak (>5 days) (*p* = 0.001).

The multivariable analysis confirmed the following predictors of RPPS: BMI (OR = 0.95, 95% CI: 0.90–0.99, *p* = 0.015), active smoking (OR = 1.64, 95% CI: 1.01–2.65, *p* = 0.044), right-sided surgery (OR = 1.83, 95% CI: 1.23–2.72, *p* = 0.003), and prolonged air leak (OR = 3.89, 95% CI: 1.60–9.84, *p* = 0.003) (Table 2).

To better evaluate the clinical implications of RPPS, a ROC curve analysis was performed to identify the cut-off point of the Collins index associated with the main postoperative complications recorded in our series-namely, prolonged air leak.

An RPPS > 10.5% according to the Collins index was significantly associated with a higher risk of prolonged (>5 days) postoperative air leak (with an AUC of 0.69, sensitivity: 69%, specificity: 54%, *p* < 0.001).

Therefore, based on this threshold, the main predictors for an RPPS with a Collins index >10.5% were explored. Univariable analysis showed the following associated factors: upper lobectomies (*p* = 0.005), lower lobectomies (*p* = 0.0017), prolonged air leak (*p* = 0.004), FVC% (*p* = 0.06), and right-sided surgery (*p* < 0.001). Right-sided surgery (OR = 2.10, 95% CI: 1.43–3.08, *p* < 0.001), upper lobectomy (OR = 1.63, 95% CI: 1.22–2.36, *p* = 0.01), and prolonged air leak (OR = 2.70, 95% CI: 1.40–5.21, *p* = 0.003) were confirmed as predictors for an RPPS > 10.5% of Collins index at multivariable analysis (Table 3).

In general, patients with RPPS also had a higher risk of postoperative subcutaneous emphysema that was only radiologically visible at the final chest X-ray (74 [15.0%] vs. 25 [5.1%], *p* = 0.041), although not clinically appreciable and not significant (25 [5.1%] vs. 7 [1.4%], *p* = 0.136). Furthermore, patients with RPPS were more likely to be discharged with a chest tube connected to a Heimlich valve (66 [13.4%] vs. 12 [2.4%], *p* < 0.001). Within 90 days of discharge, 24 patients (4.9%) were readmitted due to the increase in RPPS (7 cases [1.4%], with drainage required in 5 cases [1.0%]), worsening of subcutaneous emphysema (8 cases [1.6%], all managed conservatively with 48 h of clinical observation), and pleural effusion (9 cases [1.8%], with drainage required in 6 cases [1.2%]).

However, RPPS was not associated with a statistically significant higher risk of postoperative complications compared to the group of patients that did not have an RPSS at the final chest X-ray (71 [14.4%] vs. 30 [6.1%], *p* = 0.31), nor 90-day readmission (24 [4.9%] vs. 9 [1.8%], *p* = 0.43).

## 4. Discussion

Residual postoperative pleural space (RPPS) is a relatively common occurrence after pulmonary lobectomy, with an incidence documented in the literature ranging from 20 to 40% of cases [10,11].

Our data showed an RPPS incidence of 66.1%, seemingly higher than average, but consistent with the characteristics of our surgical population, the method used to measure RPPS, and our exclusive use of the Uniportal VATS technique [5,10]. Indeed, the use of the Collins Index in our study to quantify RPPS may partly explain the higher observed incidence. The use of an objective, accurate, and highly sensitive method such as the Collins Index, which considers the percentage of residual pleural space relative to total thoracic volume, allows for a more precise evaluation compared to traditional methods relying solely on qualitative radiographic assessments [9]. Furthermore, other intraoperative techniques and instrumentation adopted during lobectomy specific to our center could explain the results, not strictly related to the surgical approach itself (such as the location of the surgical incision and the chest tube being inserted through the same incision [5]). For instance, the completion of incomplete/absent fissures by stapler (rather than by energy devices) according to the fissure-less fissure-last technique [12,13] or the ligament division (always performed in the present series) are all factors that can impact the presence of RPPS. Unfortunately, the type of fissure (complete or incomplete) and the method used for its completion were not analyzed in this study due to its retrospective nature and lack of complete data on this variable.

The Collins method used in this study to quantify residual pleural space was validated in the literature as an effective and reproducible tool for pneumothorax measurement.

Lee et al. [14] compared the Collins [9] and Axel [15] methods for quantifying pneumothorax volume, showing that while both methods offer good accuracy, the Collins method provides more detailed and reproducible measurements, especially in cases of mild pneumothorax.

In line with the findings of Misthos et al. [3], who identified upper lobectomies, right-sided surgeries, malignant disease, and prolonged air leak as major predictors of RPPS, our multivariable analysis confirmed some of these significant predictors, such as right-sided surgery (*p* = 0.003) and prolonged air leak (*p* = 0.003) for RPPS, while upper lobectomy (*p* = 0.01) was one of the main predictors of RPPS > 10% of the Collins index in our series. The higher incidence of RPPS after upper lobectomy and right-sided resections may be explained by the specific anatomy of the upper lobes, which are less influenced by passive compensatory mechanisms such as diaphragmatic elevation and contraction of the lower intercostal muscles, and are therefore less prone to passive re-expansion after resection [16].

Additionally, the greater potential pleural space on the right side may facilitate the development of larger RPPS [16,17].

Management of prolonged air leak (PAL) is one of the key risk factors for the development of RPPS, as also highlighted by our findings. Bronstein et al. [11] emphasized that persistent PAL following pulmonary resection can prolong the duration of pleural drainage and hinder full re-expansion of the residual lung parenchyma, thus contributing to RPPS formation. Timely management of PAL—potentially involving techniques such as endoscopic closure or pleural valves—is crucial for reducing complications [11]. In our study, the use of a Heimlich valve in an outpatient setting allowed for safe management of patients with persistent but low-output PAL, avoiding invasive interventions and reducing hospital stay.

Although in most cases RPPS resolved spontaneously, a significant proportion of patients (24%) in our series were discharged with a chest drain connected to a Heimlich valve due to PAL or high-volume serous output (>500 mL/24 h). In line with the evidence-based recommendations by Korasidis et al. [16], who emphasized the importance of individualized and conservative pleural space management after lung resection, our findings confirm that RPPS often resolves without the need for invasive interventions. Moreover, Korasidis et al. [16] highlighted how the choice of surgical technique and intraoperative handling of the pleural cavity can influence the development of postoperative pleural space. While the Uniportal VATS approach offers numerous advantages in terms of reduced invasiveness [8], it does not entirely prevent RPPS, particularly in upper or right-sided lobectomies, where anatomical compensatory mechanisms are less effective [16], and the use of a single chest tube introduced through the same intercostal space as the incision in Uniportal VATS may not optimize lung inflation. Indeed, Uniportal VATS seems to be associated with a higher–although not clinically significant-incidence of postoperative RPPS [5,6,7,8,9,10] after lobectomy.

The presence of RPPS, although not associated with a higher incidence of postoperative complications in our study, can require more flexible strategies to optimize postoperative outcomes and minimize potential pleural space-related complications, as also noted by Patella et al. [17,18]. Our results showed that patients with RPPS had a higher incidence of subcutaneous emphysema visible on chest X-ray (*p* = 0.041), but not clinically significant, consistent with the findings of Thieme et al. [18], who identified RPPS as a potential air reservoir in the presence of micropleural leaks. However, these events were clinically insignificant and not associated with increased rates of complications or 90-day readmissions, as also evidenced by other studies [18].

Although RPPS itself is typically a benign and self-limiting condition, it may carry relevant logistic and management implications. Therefore, early identification of risk factors for RPPS (right-sided surgery, prolonged air leak, low BMI, active smoking) and, in particular, the identification of risk factors for RPPS > 10.5% of the Collins index (right-side surgery, upper lobectomy, and prolonged air leak), which may help in predicting PAL, allows for a more vigilant intra- and postoperative management.

In this scenario, accurate intraoperative handling of incomplete fissures [12] and checking of air leaks (with accurate intraoperative air leak measurement and aerostasis through the use of pledgeted sutures, surgical sealants as glues, or pleurodesis [19,20]) in patients at risk, as well as conservative strategies in the postoperative period, such as outpatient Heimlich valve use [18], have proved to be effective.

Future studies are warranted to identify risk factors for RPPS after Uniportal VATS lobectomy, assess the impact on postoperative outcomes and patient quality of life, and establish standardized protocols for the management and follow-up of patients with residual pleural space.

Our findings suggest that the use of Collins method can be particularly useful in clinical studies on this topic, where accurate and repeatable assessment of residual pleural space is essential.

### Limitations and Points of Strength

This study had some limitations and points of strength. First of all, it was a single-center, retrospective study with a limited series. The results obtained on the incidence of RPPS after Uniportal VATS lobectomy were not compared with those of other minimally invasive techniques or open surgery.

As a single-center study with all procedures performed by the same experienced surgical team using exclusively Uniportal VATS, our findings may have limited external validity. Therefore, our results cannot be generalized, as they may differ in other centers with different surgical expertise and different surgical techniques (such as multiport VATS or hybrid approaches).

Furthermore, the chosen cut-off of 10.5% on the Collis index to predict postoperative prolonged air leak showed a moderate discrimination power, although statistically significant (AUC 0.69, sensitivity 69%, specificity 54%).

However, a notable strength of this study was the first-time measurement of the RPPS size after pulmonary lobectomy, in particular after Uniportal VATS approach, using a standardized method commonly employed by radiologists to measure pneumothorax entity on chest X-rays (the Collins method). Moreover, all patients were operated on by the same surgical team, always adopting the same procedure with consistent intra- and postoperative management, and all underwent the same type of major anatomical resection (lobectomy). To the best of our knowledge, this is also the first study that investigated potential risk factors for the onset of RPPS volume associated with air leak and evaluated the possible clinical implications associated with RPPS after Uniportal VATS lobectomy.

## 5. Conclusions

Although RPPS is a frequent postoperative finding following Uniportal VATS lobectomy, it appears to be a benign and self-limiting condition, not associated with a higher risk of postoperative complications or 90-day readmission compared to patients who did not present RPPS. RPPS observed on the final chest X-ray was only associated with a higher risk of postoperative subcutaneous emphysema only radiologically visible but not clinically significant and with a greater likelihood of discharge with a chest tube connected to a Heimlich valve, primarily due to PAL. Our study suggests the Collins method as a valuable tool for standardizing RPPS measurement and identifies BMI, active smoking, right-sided resections, and prolonged air leak as significant predictors of RPPS. Additionally, right-sided surgery, upper lobectomy, and prolonged air leak are predictors for RPPS >10.5% on the Collins index. The identification of such factors may help clinicians in adopting early precautions–when feasible-to prevent RPPS onset and optimize postoperative management.

## Figures and Tables

**Table 1:** Clinical and preoperative characteristics of the study cohort.

	Uniportal VATS Lobectomy (#492)
**Gender (male)**	255 (51.8%)
**Age (years)**	68.62 ± 9.05
**Smoker**	120 (24.4%)
**BMI**	25.98 ± 4.49
**COPD**	132 (26.8%)
**Cardiovascular diseases**	154 (31.3%)
**Side (Right)**	301 (61.2%)
**Upper Lobectomy**	255 (51.8%)
**Lower lobectomy**	184 (37.4%)
**FEV1%**	93.81 ± 23.75
**FVC%**	104.54 ± 21.57
**DLCO**	20.96 ± 10.36
**Tumor dimension (cm)**	2.46 ± 1.41

**Table 2:** Univariable and multivariable analysis of predictors for RPPS (Bold characters = *p* < 0.05).

Variables	Univariable Analysis	Multivariable Analysis	
	*p*-Value	OR [95% CI]	*p*-Value
Age	0.208		
Sex	0.219		
BMI	**0.002**	0.95 [0.90–0.99]	**0.015**
Weight	**0.028**		
Active smoking	**0.048**	1.64 [1.10–2.65]	**0.044**
Right side	**0.003**	1.83 [1.23–2.72]	**0.033**
Upper lobectomy	0.150		
Prolonged air-leak	**0.001**	3.89 [1.60–9.84]	**0.033**

**Table 3:** Univariable and multivariable analyses for predictors of RPPS with Collins index >10.5% (Bold characters = *p* < 0.05).

Variables	Univariable Analysis	Multivariable Analysis	
	*p*-Value	OR [95% CI]	*p*-Value
Age	0.700		
Sex	0.541		
BMI	0.441		
Weight	0.986		
Active smoking	0.620		
FVC%	0.06		
Upper lobectomy	**0.005**	1.63 [1.22–2.36]	**0.01**
Lower lobectomy	**0.017**		
Right side	**<0.001**	2.10 [1.43–3.08]	**<0.001**
Prolonged air-leak	**0.004**	2.70 [1.40–5.21]	**0.003**

## Data Availability

The data presented in this study are available on request from the corresponding author.
